# A Case Report of Shingles Vaccine as a Cause of Third Nerve Palsy: A Novel Etiology

**DOI:** 10.7759/cureus.59086

**Published:** 2024-04-26

**Authors:** Jorge Sanchez, Hector DeLosSantos, Mrunalini Dandamudi, Lorenza Freddo, Guido A Macchiavello

**Affiliations:** 1 Internal Medicine, St. Barnabas Hospital Health System, Bronx, USA; 2 Neurology, St. Barnabas Hospital Health System, Bronx, USA

**Keywords:** internal medicine, neurology, zoster vaccine, vaccines, third nerve palsy

## Abstract

Vaccines are biological preparations widely used to provide acquired immunity against various life-threatening organisms and prevent severe complications of different infections. Vaccines typically demonstrate a high level of safety with minimal adverse effects. Nevertheless, it is crucial to enhance awareness when a potential new side effect emerges, as exemplified in the case discussed ahead. Despite the rarity of independent third nerve palsy occurrences, its association with the zoster vaccine remains unprecedented.

## Introduction

Zoster vaccine recombinant, adjuvanted (Shingrix), is a vaccine for adults ≥50 years of age used to prevent shingles, with its most common complication being postherpetic neuralgia. Patients are recommended to receive two doses of the vaccine at least two to six months apart [1]. The zoster vaccine is a new, adjuvanted, non-live recombinant shingles vaccine, without the capacity to produce the disease, making it less reactogenic [2]. Thus, a weaker immune response is created, and a subsequent booster injection is typically required to establish immunogenicity [3,4]. Contraindications for the vaccine include a history of severe allergic reactions (e.g., anaphylaxis) to any vaccine or vaccine component, current shingles infection, and pregnancy [1,5].

The third cranial nerve (the oculomotor nerve) innervates the eyelids, pupils, and four extra-ocular muscles: the superior rectus muscle, the medial rectus muscle, the inferior rectus muscle, and the inferior oblique muscle. These muscles, in addition to the levator palpebrae superioris and sphincter pupils, make possible most movements of the eye, such as raising the upper eyelid, looking up, looking toward the nose (adduction of the eye), and constricting the pupil. Therefore, third cranial nerve palsy will cause ptosis, ocular deviation in down and out position, and mydriasis resulting in diplopia [6-8]. This condition is described as either “third cranial nerve palsy” or “oculomotor palsy.” A post-marketing observational study noted an increased risk of developing Guillain-Barre syndrome during the first 42 days after receiving the zoster vaccine [5]; however, third cranial nerve palsy from the vaccine has not yet been described in the literature.

## Case presentation

A 69-year-old female with a past medical history of hyperlipidemia, osteoporosis, and open-angle glaucoma presented to the emergency department (ED) three weeks after receiving the zoster vaccine. She reported sharp and intermittent left-eye pain, graded at 6/10 on the pain scale, radiating to the left temporal region, and worsening when leaning forward and improving when lying down for approximately one week. In the ED, the patient’s ophthalmic examination was unremarkable. Intra-ocular pressure was within normal limits in both eyes, and the pain eventually subsided. She noted that acetaminophen provided relief from the pain. The patient was subsequently discharged to follow up with her primary care doctor.

One week later, the patient presented to the clinic complaining of an inability to move her left eye properly. On neurological examination, she had complete ptosis of the left eyelid and an inability to adduct her left eye or move it up or down. The left eye was pointed downward and outward, and the pupil was reactive to light. The right eye was within normal limits with no deficit. The patient denied pain in the area or any previous trauma, fever, or chills during the last days leading to the presentation. The rest of the neurological examination was within normal limits (no focal deficits). An isolated third nerve palsy (oculomotor nerve palsy) was diagnosed.

The patient reported that the abnormalities in her eye muscles occurred two to three days before presenting to the clinic. She did not have any active pain at that time.

A neurological evaluation was requested, and several laboratory studies and imaging were ordered, including a complete blood count, comprehensive metabolic panel, lipid panel, hemoglobin A1C, and thyroid-stimulating hormone (TSH). The results of these tests were all within normal limits.

Laboratory studies showed negative/non-reactive results for antinuclear antibodies; hepatitis A, B, and C antigens; rheumatoid factor; erythrocyte sedimentation rate; C-reactive protein; human immunodeficiency virus; measles, mumps, and rubella (titers); anti-double-stranded deoxyribonucleic acid (anti-DNA); and syphilis. However, positive results were seen for anticardiolipin antibody (AB), IgM, and ferritin, which were elevated. Additionally, varicella-zoster AB, IgM levels were elevated (Table 1). 

**Table 1:** Remarkable results. MPL: millipore luminescence

Variable	Result	Reference range
Varicella-zoster AB, IgM	1.34	0.00-0.90 index
Anti-cardiolipin AB, IgM	22	0-12 MPL U/mL
Complement component 3 (C3), serum	196 mg/dL	82-167 mg/dL
Ferritin	255 ng/mL	12-150 ng/mL

**Figure 1:**
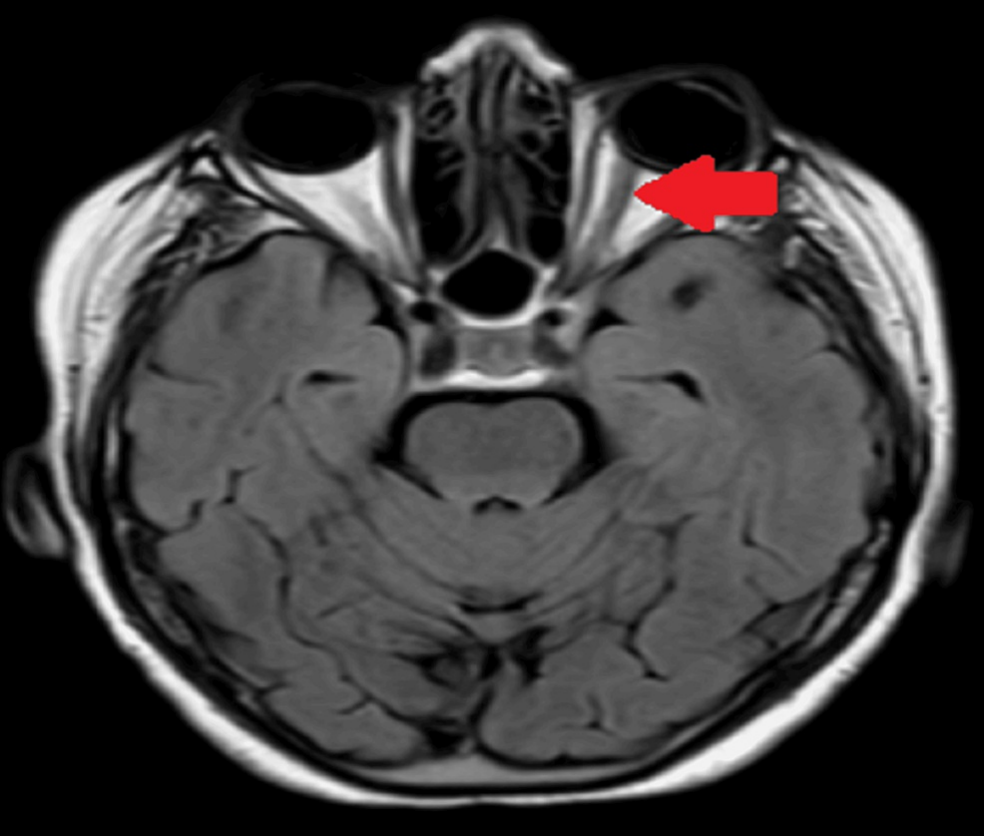
Brain magnetic resonance imaging shows intact brain anatomy and intact optic nerves.

**Figure 2:**
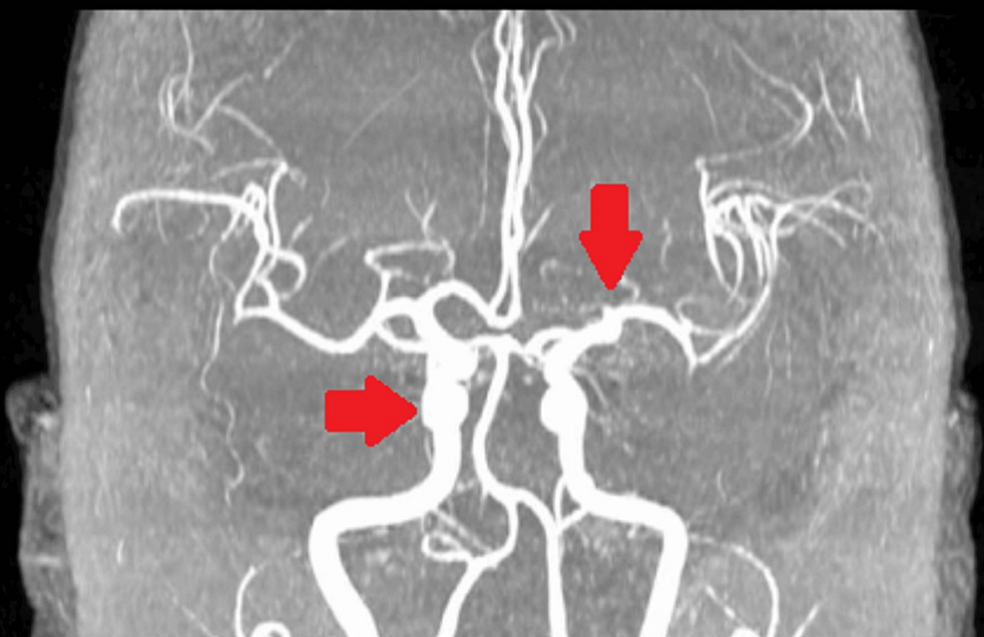
Brain magnetic resonance angiography shows normal brain vasculature without contrast highlighting (i.e., no aneurysm or mass).

Subsequently, brain magnetic resonance imaging and brain magnetic resonance angiography were performed to rule out any anatomic cause of the current illness that would explain the symptoms (Figures 1-2). However, imaging did not show any space-occupying lesions or aneurysms.

Following neurology recommendations, a lumbar puncture was performed. CSF glucose, CSF protein, cell count, protein electrophoresis, and oligoclonal banding were all either negative or within normal limits. Given the results, as well as the patient’s history and physical examination, the diagnosis of isolated third nerve palsy, most likely secondary to the zoster vaccine, was made. The patient was subsequently started on a seven-week prednisone taper. By the fourth week of treatment, the patient’s palsy was mainly resolved, and she was completely asymptomatic by the end of the taper.

It is important to note that the ambulatory care clinic has followed the patient for more than 13 years, with all chronic conditions controlled and within the range of medical guidelines.

## Discussion

The most common side effects of the zoster vaccine can be either localized or generalized. The most common localized side effects include pain (78% of cases), redness (38% of cases), and swelling (26% of cases) at the site of injection [5], and the most common generalized side effects include myalgia (45% of cases), fatigue (45% of cases), headache (38% of cases), fever (21% of cases), and gastrointestinal symptoms (17% of cases).

Third cranial nerve palsy manifests clinically as the patient’s affected eye looking “outward and downward” as a result of the unopposed action of the lateral rectus and superior oblique muscle, causing the classic presentation of this palsy [6,9]. Third cranial nerve palsy can be classified into two different types based on pupil reactivity. The first type is pupil-sparing third nerve palsy, which is due to micro-vascular or systemic causes (e.g., hypertension, diabetes mellitus, and hyperlipidemia). The second type is non-pupil-sparing palsy usually secondary to nerve compression, which should immediately prompt a neurological evaluation that includes imaging studies to rule out any intracranial anomaly (e.g., tumor or aneurysm), which are common etiologies of the condition [10-13]. The prognosis for a full recovery is very promising when suffering from third nerve palsy due to a microvascular etiology, as most cases recover completely within three to six months, and almost all cases (90%) recover within 12 months [14,15].

A study conducted by Galtrey et al. showed that risk factors for microvascular etiology include age (93% of cases being >50 years old), hypertension (25% of cases), diabetes (18% of cases), or both hypertension and diabetes (7% of cases), of which our patient only had one (i.e., age) [14,16]; she did not have any other obvious risk factors for a possible systemic etiology. In addition, her blood pressure and hemoglobin A1C level were always within normal limits during all previous visits (>13 years, with all chronic conditions controlled and within the range of medical guidelines).

Our patient’s palsy spared the pupil; in such cases, it is unusual for it to be due to mass effect. However, there have been reported cases in which this type of palsy results from some mass compressing the nerve intracranially, as presented in the study by Fang et al. [11], which is why brain imaging is always performed to rule it out.

Mouton and Meyer presented a case report in which the patient had a non-pupil-sparing third nerve palsy, which precipitated existing glaucoma in the patient, making it an acute ophthalmology emergency secondary to the pupillary dilation [16,17]. Such a presentation is rare, as these palsies are rarely an emergency.

A few cases of isolated third nerve palsy had been linked to hyperlipidemia, as the case was reported by Gupta [18]. The patient had hyperlipemia with a triglyceride level of 630 mg/dL and a low-density lipoprotein (LDL) level of 135 mg/dL and received treatment as per the lipid association guidelines with lipid-lowering agents, as well as anti-inflammatory medication for possible inflammation and pain [18]. In our case, the patient had her lipid profile under control when the symptoms presented. She had a triglyceride level of 154 mg/dL and an LDL level of 88.2 mg/dL, which were ideal for her age and co-morbidities as per American College of Cardiology/American Heart Association lipid management guidelines [19], effectively ruling this out as a possible cause of the palsy.

Third cranial nerve palsy has not been associated with the zoster vaccine. However, it has been associated with other types of vaccines in the past, as seen by Kerbage et al., where palsy was linked to a COVID-19 vaccine [20]. The pathophysiology behind it is not well described and is currently uncertain. Nonetheless, it is hypothesized that it could be due to an immunomodulatory response of the cells, causing damage to the peripheral nerve neurons at the cellular level, and affecting the myelin sheaths and surrounding axons [20].

The Naranjo Adverse Drug Reaction (ADR) Probability Scale was performed. The patient scored a 6, indicating that the vaccine is likely responsible for the ADR in this case. It cannot be classified as a definite ADR because the drug cannot be established in body fluids or tissues, and no reexposure was performed [21].

## Conclusions

This case makes us look further into the possible etiologies of the pathology of third nerve palsy, keeping us on high alert for this type of situation and possible side effects of commonly used drugs such as vaccines. We aim to raise awareness about this potential but uncommon side effect of the zoster vaccine. Therefore, this case will be reported to the Vaccine Adverse Event Reporting System.

## References

[1] (2022). Centers for Disease Control and Prevention: Shingles vaccination. https://www.cdc.gov/vaccines/vpd/shingles/public/shingrix/index.html.

[2] (2022). Centers for Disease Control and Prevention: Shingrix vaccine FAQs. https://www.cdc.gov/vaccines/vpd/shingles/hcp/shingrix/faqs.html.

[3] (2022). US Department of Health and Human Services: vaccine types. https://www.hhs.gov/immunization/basics/types/index.html.

[4] Nunnally BK, Turula VE, Sitrin RD (2014). Vaccine Analysis: Strategies, Principles, and Control. https://link.springer.com/book/10.1007/978-3-662-45024-6.

[5] Dooling KL, Guo A, Patel M, Lee GM, Moore K, Belongia EA, Harpaz R (2022). Recommendations of the Advisory Committee on Immunization Practices for use of herpes zoster vaccines. MMWR Morb Mortal Wkly Rep.

[6] Joyce C, Le PH, Peterson DC (2022). Neuroanatomy, cranial nerve 3 (oculomotor). StatPearls [Internet].

[7] Merck Manual: Third Cranial Nerve (Oculomotor Nerve) Palsy—Brain, Spinal Cord, and Nerve Disorders. (n.d.). Merck Manuals Consumer Version (2022). Third cranial nerve (oculomotor nerve) palsy-brain, spinal cord, and nerve disorders. November 12, 2022.

[8] Wilson-Pauwels L, Stewart PA, Akesson EJ (2022). Cranial Nerves: Function and Dysfunction. https://pmphusa.com/book/cranial-nerves-function-and-dysfunction/.

[9] Yang Y, Lai C, Yan F, Wang J (2020). Clinical significance of MRI contrast enhancement of the oculomotor nerve in ischemic isolated oculomotor nerve palsy. J Clin Neurol.

[10] Modi P, Arsiwalla T (2022). Cranial nerve III palsy. StatPearls [Internet].

[11] Fang C, Leavitt JA, Hodge DO, Holmes JM, Mohney BG, Chen JJ (2017). Incidence and etiologies of acquired third nerve palsy using a population-based method. JAMA Ophthalmol.

[12] Lemos J, Eggenberger E (2015). Neuro-ophthalmological emergencies. Neurohospitalist.

[13] Goldstein JE, Cogan DG (1960). Diabetic ophthalmoplegia with special reference to the pupil. Arch Ophthalmol.

[14] Galtrey CM, Schon F, Nitkunan A (2015). Microvascular non-arteritic ocular motor nerve palsies-what we know and how should we treat?. Neuroophthalmology.

[15] Canady FJ, Ricca AM, Stiff HA, Shriver EM, Ko AC (2022). Self-resolving ischemic third nerve palsy. https://webeye.ophth.uiowa.edu/eyeforum/cases/280-self-resolving-ischemic-TNP.htm.

[16] Mouton DP, Meyer D (1989). Oculomotor nerve palsy precipitating acute angle-closure glaucoma. A case report. S Afr Med J.

[17] Jacobson DM, McCanna TD, Layde PM (1994). Risk factors for ischemic ocular motor nerve palsies. Arch Ophthalmol.

[18] Gupta D (2022). Isolated oculomotor palsy secondary to dyslipidemia. J Neurol Stroke.

[19] Reiter-Brennan C, Osei AD, Iftekhar Uddin SM (2020). ACC/AHA lipid guidelines: personalized care to prevent cardiovascular disease. Cleve Clin J Med.

[20] Kerbage A, Haddad SF, Haddad F (2022). Presumed oculomotor nerve palsy following COVID-19 vaccination. SAGE Open Med Case Rep.

[21] Naranjo CA, Busto U, Sellers EM (1981). A method for estimating the probability of adverse drug reactions. Clin Pharmacol Ther.

